# Hepatorenal Syndrome—Novel Insights into Diagnostics and Treatment

**DOI:** 10.3390/ijms242417469

**Published:** 2023-12-14

**Authors:** Krzysztof Badura, Weronika Frąk, Joanna Hajdys, Gabriela Majchrowicz, Ewelina Młynarska, Jacek Rysz, Beata Franczyk

**Affiliations:** 1 Department of Nephrocardiology, Medical University of Lodz, Ul. Zeromskiego 113, 90-549 Lodz, Poland; 2 Department of Nephrology, Hypertension and Family Medicine, Medical University of Lodz, Ul. Zeromskiego 113, 90-549 Lodz, Poland

**Keywords:** hepatorenal syndrome, cirrhosis, acute kidney injury, pathophysiology, biomarkers, vasoconstrictor drugs, liver transplantation, novel therapies

## Abstract

Hepatorenal syndrome (HRS) is a disorder associated with cirrhosis and renal impairment, with portal hypertension as its major underlying cause. Moreover, HRS is the third most common cause of acute kidney injury, thus creating a major public health concern. This review summarizes the available information on the pathophysiological implications of HRS. We discuss pathogenesis associated with HRS. Mechanisms such as dysfunction of the circulatory system, bacterial infection, inflammation, impaired renal autoregulation, circulatory, and others, which have been identified as critical pathways for development of HRS, have become easier to diagnose in recent years. Additionally, relatively recently, renal dysfunction biomarkers have been found indicating renal injury, which are involved in the pathophysiology of HRS. This review also summarizes the available information on the management of HRS, focusing on vasoconstrictive drugs, renal replacement therapy, and liver transplant together with currently being investigated novel therapies. Analyzing new discoveries for the underlying causes of this condition assists the general research to improve understanding of the mechanism of pathophysiology and thus prevention of HRS.

## 1. Introduction

Cirrhosis is a major cause of morbidity and mortality in people with chronic liver disease worldwide [1]. It evolves from an asymptomatic phase (compensated cirrhosis) to a symptomatic phase (decompensated cirrhosis). In its compensated state, persistence of liver damage results in increasing fibrosis and portal hypertension. Portal hypertension is the key factor leading to the shift from compensated to decompensated cirrhosis. Further decompensation events such as refractory ascites, spontaneous bacterial peritonitis, hepatorenal syndrome (HRS), recurrent hepatic encephalopathy, and variceal bleeding further reduce survival [2,3]. HRS, in particular, has an extremely poor prognosis, necessitating early identification and understanding of the syndrome [4].

Renal impairment is a severe complication in patients with advanced cirrhosis [5]. The HRS is characterized by renal failure and major disturbances in circulatory function. The HRS is a diagnosis of exclusion and occurs with equal frequency in both sexes [4,6]. The incidence of HRS varies from 7% to 45% [7,8,9,10]. However, despite adequate treatment mortality is still about 60% and higher [11,12]. Moreover, in accordance with research conducted by Patel et al. [13], HRS is associated with higher health care utilization and cost burden compared to cirrhosis alone, highlighting the importance for improved screening and treatment approaches. It is divided into two types, in accordance with guidelines of the International Club of Ascites (ICA) [14,15,16,17]. The first represents an acute impairment of kidney function, HRS-AKI (acute kidney injury), whereas the second represents a more chronic kidney dysfunction, HRS-CKD (chronic kidney disease). Types of HRS are summarized in Table 1. HRS is diagnosed through a decline in kidney function, with no presence of underlying kidney diseases like hematuria or proteinuria. HRS can potentially be reversed with either liver transplant or vasoconstrictor medications. The progression and severity of symptoms vary between the two types, with HRS-AKI being notably more severe and characterized by a rapid progression [18,19]. HRS-AKI is regarded as the most common fatal complication in patients with cirrhosis. Nevertheless, HRS is associated with poor prognosis with high mortality rates, notably in individuals with advanced cirrhosis [4,14].

The purpose of this paper is to present the advances in understanding and managing HRS. We describe the definition, etiology, and pathogenesis of HRS. We additionally discussed renal dysfunction biomarkers such as serum creatinine, cystatin C, tubular injury biomarkers, and others together with differential diagnosis. Moreover, we summarized the medical interventions used in managing HRS, particularly novelty therapies. However, further investigation is crucial to improve our knowledge of HRS progression and, more importantly, accelerate basic research to improve our understanding of the mechanism of pathophysiology.

## 2. Pathogenesis of the Onset of Hepatorenal Syndrome

The pathogenesis of HRS is intricate and results from the interaction of numerous pathophysiological mechanisms in cirrhotic patients. Among the most important underlying causes for the development of HRS are circulatory dysfunction [20], the influence of circulating inflammatory mediators, bacterial infection [21], impaired renal autoregulation and renal tubular function [22], the occurrence of hepatorenal reflex, adrenal insufficiency, involvement of bile acids, or intra-abdominal hypertension [11].

On the basis of observations, circulatory dysfunction resulting from portal hypertension and cirrhotic cardiomyopathy in advanced cirrhosis is thought to play a key role in the occurrence of HRS [23,24,25,26,27,28].

According to the theory of arterial vasodilation, portal hypertension occurring in the course of cirrhosis contributes to excessive production and activation of vasodilator factors such as nitric oxide (NO), prostacyclins, carbon monoxide, and endogenous endocannabinoids by hepatocytes and stellate cells [24,29,30,31,32]. Local vasodilation of splanchnic blood vessels leads to a reduction in vascular resistance and therefore in effective arterial blood volume (EABV) and blood pressure (BP) [23,29,33]. In the initial stages of the disease, this effect is compensated for by an increase in cardiac output (CO), whereas in the advanced stages of cirrhosis, CO is no longer able to maintain adequate values and begins to decline due to myocardial overload [20,23,31,34]. One of the reasons for this is the effect of the developing cirrhotic cardiomyopathy, which through constant stimulation of the renin-angiotensin-aldosterone system (RAAS) and the sympathetic nervous system (SNS) results in an impaired myocardial response to stress, diastolic dysfunction, and electrophysiological abnormalities such as QT interval prolongation [29,35,36,37].

There is a decrease in systemic vascular resistance, hypovolemia, and hypotension due to the resulting disproportion between intravascular blood volume and dilated blood vessels [25,31]. In response to the above condition, high-pressure baroreceptors in the carotid body and aortic arch are unloaded leading to activation of the RAAS, the SNS and the release of vasopressin to maintain EABV and increase CO and heart rate (HR) [6,24,26,31,32,37,38]. Nevertheless, in the long term, these mechanisms have detrimental effects on renal function by increasing sodium and water retention and constricting renal vessels leading to reduced renal blood flow [39,40,41].

The decreased renal blood flow is further caused by increasing ammonia concentrations, which contribute to reduced nitric oxide production in the renal microcirculation by interfering with arginine metabolism [42]. Regardless of the reduced blood flow, the kidneys initially maintain an adequate glomerular filtration rate (GFR) due to prostaglandins I2 and E2, both of which have a protective role by exerting a dilatory effect on afferent arterioles, thereby counteracting the effects of increased RAAS and SNS activity on the renal circulation [43,44]. However, with the progression of liver disease, this imbalance deteriorates and a progressive decline in GFR occurs [26]. In addition, renal autoregulation is impaired in HRS patients, resulting in lower renal blood flow at a given arterial pressure than at the same pressure in healthy individuals, and this is associated with an increased risk of developing AKI [45,46].

Patients with uncompensated cirrhosis have been observed to have a persistent, elevated systemic inflammatory response, despite the absence of overt infection, which contributes to disease progression and is additionally associated with complications such as HRS [47,48,49]. Patients are characterized by elevated serum levels of C-reactive protein and increased leukocyte levels, which increase with disease progression [50,51,52]. Moreover, increased levels of pro-inflammatory cytokines such as IL-6, IL-8, TNF-α, vascular cell adhesion molecule-1 (VCAM-1) are found in the plasma of these patients and increased levels of monocyte chemoattractant protein-1 are observed in the urine [47,48,53,54,55].

The occurrence of bacterial infection in cirrhotic patients significantly increases their risk of developing HRS. It most frequently evolves in patients who have had spontaneous bacterial peritonitis [56,57,58,59,60,61]. However, any infection in a cirrhotic patient should be treated and each patient should be monitored for the potential evolution of HRS [49,57,62]. It is important to remember that patients with cirrhosis do not necessarily have an overt bacterial infection to develop a generalized inflammatory response.

In patients with cirrhosis, bacterial translocation from the gastrointestinal tract to the mesenteric lymph nodes has been shown to contribute to increased release of pro-inflammatory cytokines [63,64]. Pathogen-associated molecular patterns (PAMPs), which are fragments of bacteria from translocation or from bacterial infection, including lipopolysaccharides, flagellin, and nigericin and are one of two groups of molecules associated with systemic inflammation in patients with cirrhosis. The second group are damage-associated molecular patterns (DAMPs), such as high mobility group B1 protein (HMGB1), heat shock protein (HSP), adenosine triphosphate (ATP), or double-stranded genomic DNA, which are released from damaged hepatocytes [65,66,67,68]. Both groups of molecules can activate pattern recognition receptors present on circulating innate response immune cells such as TLRs, leading to the activation of these cells and the release of pro-inflammatory cytokines [48,69,70].

Increased levels of pro-inflammatory cytokines affect vascular smooth muscle and are concomitantly associated with increased release of vasodilator factors such as NO or CO, consequently leading to a further decrease in vascular resistance and EABV. This is associated with further circulatory dysfunction in cirrhotic patients resulting in the development of HRS [47,59,71,72].

In addition to their inflammatory effects, PAMPs and DAMPs have direct effects on the kidneys, as increased expression of TLR4 and caspase 3 receptors in renal tubular cells is observed in cirrhotic patients [22,70]. This may be due to direct exposure to PAMPs, but it is also thought that TLR4 expression is increased as a result of renal ischemia caused by reduced renal blood flow [73,74,75]. Upregulation of these receptors in patients is associated with the development of severe renal dysfunction through renal tubular damage and apoptosis [64,70,73].

Furthermore, the co-occurrence of inflammation and renal circulatory dysfunction results in reduced renal tubular cell metabolism and altered cell function, favoring cell survival over other functions. There is impaired absorption of sodium and chloride, whose increased concentration, sensed by the macula densa, causes further activation of the RAAS system, leading to a decrease in GFR [15,76].

Relative adrenal insufficiency (RAI) has been found in 25–65% of patients with decompensated cirrhosis, including 80% of those with coexisting cirrhosis and HRS. It may therefore play an important role in the development of HRS [19,77,78,79,80]. Patients with RAI have lower blood pressure and higher serum renin and norepinephrine levels, thus increasing the risk of AKI in HRS and sepsis [81,82,83,84,85]. Although the mechanisms are not fully understood, it is thought to be related to depletion of substrates necessary for cortisol synthesis, inadequate adrenal blood supply due to arterial vasoconstriction, adrenal injury due to coagulopathy, and disruption of the hypothalamic-pituitary axis by PAMPs and pro-inflammatory cytokines [81,86,87].

A number of studies have demonstrated the involvement of cholestatic nephropathy in the evolution of AKI-HRS in patients with cirrhosis and high serum bilirubin levels, which has been linked to the formation of intra-renal bile acid casts, which have shown direct toxicity to renal tubular cells [88,89,90].

In patients with refractory ascites, the elevated intra-abdominal pressure that occurs may be a cause of AKI and contribute to the development of HRS [91,92]. Persistent intra-abdominal pressure above 20 mmHg has been shown to be associated with organ dysfunction, out of which renal damage occurs earliest [93]. Potential mechanisms of impaired renal function include reduced renal blood flow and renal parenchymal compression associated with persistently increased intra-abdominal pressure. Moreover, increased renal vascular resistance in combination with other factors such as reduced CO, elevated RAAS, catecholamines, and inflammatory cytokines leading to impaired glomerular and tubular renal function [94,95,96]. It has been shown that performing paracentesis in such patients is associated with a significant improvement in creatinine clearance. However, it should be noted that performing paracentesis can potentially induce circulatory dysfunction and therefore such an intervention will not have the intended effect on everyone [97,98].

The development of HRS is also thought to involve a direct link between the liver and the kidneys through the presence of osmoreceptors, chemoreceptors, and baroreceptors in the liver, which directly influence renal function through neural connections [99,100]. Experimental studies have shown that reduced portal vein blood flow stimulates intrahepatic adenosine receptors, resulting in renal sodium and water retention. A similar effect has been observed in patients with HRS [101]. Furthermore, studies have shown that patients with cirrhosis develop a disturbance in liver architecture associated with changes in sinusoidal pressure, which stimulates the release of adenosine. Neuronal connections then lead to renal vasoconstriction and reduced renal blood flow [101,102].

**Figure 1:**
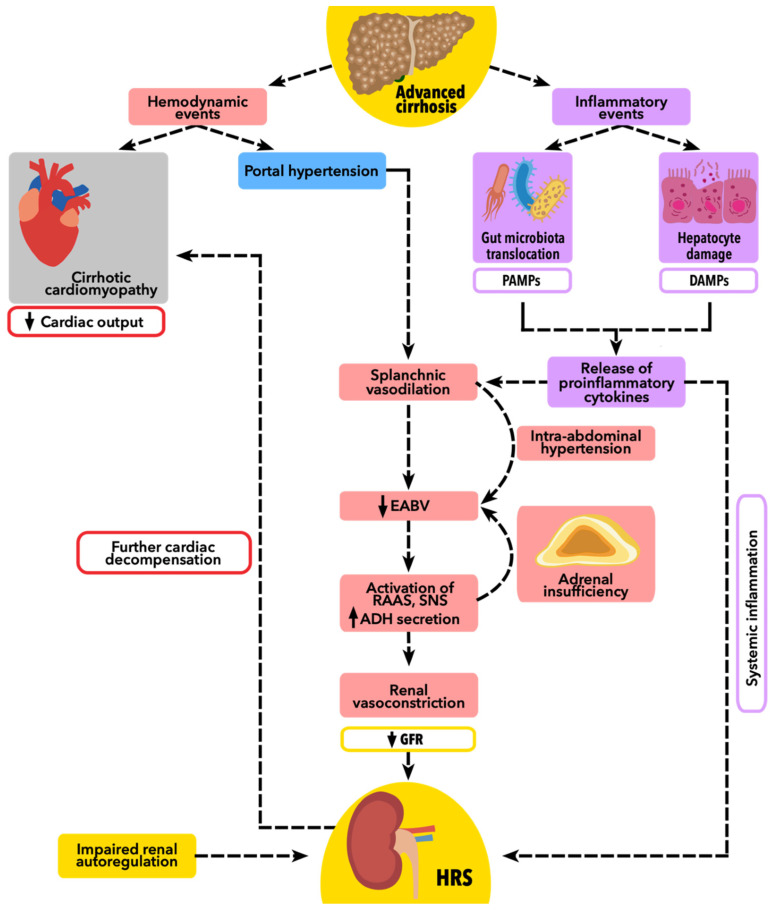
Pathophysiological mechanisms resulting in the development of HRS. Abbreviations: PAMPS—pathogen-associated molecular patterns; DAMPs—damage-associated molecular patterns; EABV—effective arterial blood volume; RAAS—renin-angiotensin-aldosterone system; SNS—sympathetic nervous system; ADH—antidiuretic hormone; GFR—glomerular filtration rate; HRS—hepatorenal syndrome.

The mechanisms contributing to the development of HRS mentioned above are presented in Figure 1.

## 3. Diagnosis

### 3.1. Renal Dysfunction Biomarkers in Patients with Impaired Liver Function

Currently available biomarkers that are useful in the diagnostic process of renal dysfunction can be subdivided into three groups: functional biomarkers, tubular injury biomarkers, and biomarkers of cell cycle arrest [103].

#### 3.1.1. Functional Biomarkers—Glomerular Filtration Rate Estimation

##### Serum Creatine

According to recent KDIGO guidelines for AKI and ICA consensus, serum creatinine (sCr) is a crucial biomarker to diagnose a decreased renal function among patients with or without liver dysfunction [104,105]. Reduced GFR due to the kidney dysfunction can be easily revealed by sCr in both cirrhotic and non-cirrhotic patients; however, several studies have shown, that impaired liver function can decrease sCr levels [104]. As a consequence, sCr measurements in patients with cirrhosis may lead to overestimation of eGFR and renal function.

The decrease in sCr levels in patients with cirrhosis may be caused by various disorders at once. Creatinine is a product of creatinine phosphate breakdown in muscle. Levels of sCr in stable kidney function and muscle weight often remain constant due to the production–excretion balance. Creatinine is constantly produced in skeletal muscles, with the production rate depending on the absolute muscle weight [106]. Stabile sCr levels are remained by constant glomerular filtration of creatinine and its excretion in proximal tubule of the nephron [106]. Due to the absence of tubular reabsorption of creatinine, its clearance can be used for glomerular filtration rate estimation [107]. One of the most important mechanisms in sCr decrease is a muscle weight loss that ranges from 40–70% among patients with cirrhosis [108]. Main pathophysiological factors contributing to anabolic resistance leading subsequently to muscle weight loss are cytokine release due to the hepatocellular necrosis; increase in PAMPs and DAMPs; portosystemic collateral circulation causing hyperammonemia and endotoxemia; and underlying etiology of liver disease (ethanol, cholestasis, insulin resistance) [108]. However, muscle weight loss seems to be a reasonable explanation for decline creatinine formation and subsequent decreased sCr levels, liver cirrhosis may lead to increased excretion of creatinine caused by its increased tubular secretion [104]. The most likely hypotheses explaining the increased creatinine excretion within the renal tubules are hyperuricuria and polyuria in cirrhotic patients that may suggest the presence of tubular damage leading to the leakage of creatinine [109]. Moreover, increased tubular secretion of creatinine correlates with decreased GFR among patients with cirrhosis, which may significantly increase the underestimation of eGFR from the sCr-based formula [109]. Another problem that occurs when estimating kidney function using sCr levels is an interference between bilirubin and creatinine, that occur in Jaffe reaction—the most common method to assess sCr in clinical practice [110,111]. It is possible to minimalize the risk of sCr underestimation in Jaffe method by serum deproteinization or “rate-blanking”. Due to the lack of automatization, serum deproteinization method cannot be used routinely, whereas “rate-blanking” method is not currently available for all of the reagents used in Jaffe method [111]. To avoid the bilirubin-creatinine interference, the reagents based on enzymatic methods can be used; however, the interference and subsequent renal function overestimation still should be considered [111]. Finally, decreased level of sCr among cirrhotic patients can be explained by overhydration resulting in sCr dilution [109].

##### Cystatin C

In recent years, a method to determine kidney function that has become increasingly popular, especially when estimating GFR using sCr may be at risk of bias, is to calculate GFR using the Cystatin C level [103,112]. Cystatin C is non-glycolisated protein produced by all human nucleated cells, filtered in glomeruli without further secretion in proximal renal tubule [113]. In contrast to creatinine, cystatin C level has no correlation with muscle mass that can be important asset among cachectic patients [103,112,113]. Moreover, Cystatin C is less affected by age and race, and it is a more reliable biomarker in declined eGFR between 60–90 mL/min/1.73 m^2^, whereas significant changes in sCr are rarely observed [113]. Recent studies have shown that cystatin C does not overestimate renal function in cirrhosis as much as sCr and is a more accurate biomarker of renal dysfunction in patients with depleted liver function [114,115,116]. However, while Cystatin C-based estimation of GFR seems to be highly desirable in patients suffering from cirrhosis, it should be noted that there are several factors modifying both cystatin C and sCr levels [117,118]. The awareness of functional biomarkers limitations seems to be crucial when choosing a method for GFR estimation and for further proper renal function assessment [117,118]. Summarized characteristics of presented renal functional biomarkers are presented in Table 2.

#### 3.1.2. Tubular Injury Biomarkers and Diagnostic Methods

In recent years, many studies focused on tubular injury biomarkers that may be indispensable tool for differential diagnosis in patients presenting with acute kidney dysfunction. Among the many studied makers important in the differential diagnosis of AKI, the most useful seem to be Interleukin 18 (IL18), neutrophil gelatinase-associated lipocalin (NGAL), liver fatty-acid binding protein (L-FABP), and kidney injury molecule (KIM-1) [103,122].

##### Interleukin 18

IL18 is expressed as an inactive precursor mainly in interstitial macrophages, proximal tubule, and collecting duct cells, and it remains intracellular until its breakdown mediated by caspase-1, a component of inflammasome. Inflammasome is a protein complex mediating the cleavage and further release of interleukins in response to extrinsic cell injury, including renal tubule damage [123]. Although IL18 release often indicate renal tubular cells damage, it is noteworthy that IL18 is constantly produced in intercalated cells of collecting duct in healthy kidney [123]. Several studies have shown that in patients with cirrhosis, urinary IL18 concentrations vary depending on the type of the kidney injury. Values of urinary IL18 are significantly higher in patients with CKD, prerenal AKI, and HRS whereas significantly lower in comparison with ATN patients [124]. Values of urinary IL18 should be interpreted cautiously, due to some limitations caused by extrarenal factors that are not associated with ATN, such as sepsis, inflammation, urinary tract infections, and ischemia-reperfusion injury that may increase urinary levels of this biomarker [125,126,127].

##### NGAL

NGAL belonging to lipocain family exist in the human body in two forms: monomer and homodimer [123]. The monomer is a direct product of gene expression with possible further homodimerization in neutrophils [123]. NGAL present in serum is filtrated in renal glomeruli and subsequently reabsorbed in proximal tubule [123]. In kidneys, it is mostly expressed in ascending loops of Henle and in intercalated cells of collecting duct [123]. Secondary to the renal epithelial cell injury or stimulation, NGAL can be rapidly secreted causing elevated concentrations in urine indicating tubular-cell damage [123]. However, NGAL is a biomarker of tubular injury, some studies suggest that elevated urinary NGAL can predict the HRS in cirrhotic patients with normal sCr levels [128]. In patients with elevated NGAL and normal sCr values, urinary tract infections should be considered, that can be the cause of increased levels of urine NGAL [123]. Interestingly, several studies have shown that volume status, diuretics, and prerenal AKI have no influence on urinary NGAL, and that increase its utility in differential diagnosis [129]. Moreover, high urinary NGAL levels on hospital admission are independently associated with poor prognosis [130]. Finally, it should be noted that highest values of urinary NGAL were observed in patients with ATN-AKI, intermediate in HRS-AKI, and low in prerenal-AKI, whereas NGAL is thought to have the highest predictive accuracy for ATN diagnosis [130,131].

##### L-FABP

The family of lipid-binding proteins comprise tissue-specific proteins named after the tissue where they first have been discovered [123]. This protein family is mainly responsible for regulatory functions of fatty acid uptake and intracellular transport, whereas L-FABP binds fatty acids and transports them to mitochondria and peroxisomes where energy for tubular cells is produced [123]. Moreover, L-FABP expression can be induced by hypoxia and protect cells from oxidative stress injury [123]. In kidneys, L-FABP is located similarly as N-GAL in proximal tubule, and it is excreted secondary to tubular cell damage [123]. Several studies have shown that in patients with liver cirrhosis, NGAL can be useful biomarker in differential diagnosis. Belcher et al. [132] has shown statistically significant difference in L-FABP values among patients with prerenal-AKI, ATN-AKI, and HRS-AKI. Patients with ATN-AKI had the highest values of L-FABP, whereas in HRS-AKI only intermediate elevation has been observed [132]. The lowest values of L-FABP were among patients with prerenal-AKI [132]. Moreover, it has been observed that subsequent HRS development occurred more often in individuals with higher values of L-FABP at baseline, However, no statistically significant association has been confirmed [128].

##### KIM-1

KIM-1 is a transmembrane glycoprotein expressed at low levels in kidneys and other organs [123]. Secondary to ischemia-reperfusion or toxic injury, the expression of KIM-1 escalates mainly in proximal tubule [123]. Studies on animal models suggest that KIM-1 can have an important role in kidney protection and recovery, where KIM-1 acts like phosphatidylserine and mediates the process of tubular cell phagocytosis [123]. It has been confirmed that KIM-1 can be useful in differential diagnosis, where urine levels of that biomarker are significantly higher among patients with ATN-AKI [132]. Furthermore, slightly elevated urine KIM-1 can be revealed in individuals with HRS-AKI, whereas no significant elevation among patients with prerenal AKI has been observed [132]. Therefore, the utility of KIM-1 to distinguish ATN-AKI from HRS-AKI may be limited. Another study suggesting potential usefulness of KIM-1 measurements in differentiating prerenal-AKI, HRS-AKI, and ATN-AKI has shown increased KIM-1 among patients with ATN-AKI and HRS-AKI [131]. No difference and KIM-1 elevation between the group without AKI and prerenal AKI has been observed [131]. Highest KIM-1 levels have been observed among patients with ATN-AKI, whereas patients with HRS-AKI had only moderately increased values [131]. Interestingly, similar to N-GAL, increased KIM-1 can predict the development of HRS-AKI among individuals with liver cirrhosis and can be associated with adverse patient outcomes [128].

##### Other ATN Biomarkers

Trefoil Factor 3 (TFF-3) is a small peptide hormone mainly secreted by epithelial cells in gastrointestinal tract [133]. In kidneys, TFF-3 is expressed in collecting duct cells, However, the role of TFF3 remains unknown [133]. TFF-3 levels are increased in serum as well as in urine secondary to CKD, therefore it is suggested that TFF-3 may be useful in differential diagnosis of AKI [131,133]. Measurements of TFF-3 can be useful in AKI diagnosis, where, in comparison to non-AKI patients TFF-3 is often significantly increased. Moreover, levels of urinary T FF-3 vary on the AKI type—patients with prerenal or HRS-AKI have moderately increased TFF-3, whereas when ATN-AKI occurs, TFF-3 levels are highly increased [131]. Noteworthy, TFF-3 levels are influenced by the liver function, where acute-on-chronic liver failure significantly increase urinary TFF-3 [131].

Another promising yet poorly studied biomarker to differentiate HRS-AKI from ATN-AKI is calbindin [131,134]. Calbindin is an intracellular calcium-binding protein produced in the kidney, gastrointestinal tract, brain, and pancreas that take part in calcium homeostasis regulation [134]. In one study, it has been confirmed that urinary levels of calbindin were significantly higher in patients with ATN, whereas lower in patients with prerenal AKI or HRS-AKI when compared to non-AKI patients [131]. It should also be noted that acute-on-chronic liver failure did not increase urinary calbindin levels that could increase usefulness among patients with advanced liver disease [131].

Glutathione-S-transferase-π (GST-π) belongs to glutathione-transferases (GSTs) family that is highly involved in detoxification processes of hydrophobic and electrophilic molecules [135]. GSTs catalyse the intracellular conjugation of xenobiotics and carcinogens with glutathione that allows toxin elimination through the kidneys [135]. Considering the tubular injury diagnosis, it should be noted that in kidney GST-α and GST-π are highly specific to proximal and distal tubule epithelium, respectively [136,137,138]. In recent years, several studies have shown that GST-π may be a valuable tool for AKI diagnosis and its prognosis assessment [136,139]. Currently available data suggest that urinary GST-π can be useful in differentiating between AKI types and can be challenging in patients with suspected HRS-AKI; however, scientific evidence is highly limited. Among patients with liver cirrhosis there was no significant association between GST-π levels and AKI presence or absence; however, GST-π values were highly increased in patients with ATN-AKI [131]. Furthermore, there were no differences in GST-π between no AKI, prerenal AKI and HRS-AKI patients [131]. Therefore, GST-π may be concerned as a biomarker useful in distinguishing between ATN-AKI and other types, whereas its predictive accuracy for ATN diagnosis is lower when compared to NGAL [131]

In comparison to the biomarkers presented above, osteopontin (OPN) seems to be better explored molecule with multiple functions in human body. OPN belongs to small integrin binding ligand N-linked glycoprotein (SIBLING) family that is involved in the process of bone mineralization, nevertheless it is also expressed in various immunological cells such as activated T cells, macrophages, natural killer (NK) cells, and much more [140]. To sum up, OPN is not only involved in bone-associated reactions, but also with regulation of immune response and can be helpful in autoimmune disease diagnostics [140]. In kidneys, OPN is expressed in ascending limb of the loop of Henle as well as in collecting ducts [140]. The role of OPN in the human kidney remains unclear; however, some studies indicate its potential influence on the embryonic kidney formation processes [140]. Recent studies suggest that OPN can be useful in AKI diagnosis and prognosis estimation [131,141,142,143]. According to acute kidney dysfunction among cirrhotic patients, levels of urinary OPN vary depending on the AKI subtypes [131]. Apart from non-AKI patients, the lowest OPN values have been observed among prerenal AKI patients, whereas intermediate elevation in HRS-AKI and the greatest elevation ATN-AKI have been observed [131]. It should be noted that some factors modify OPN expression, where parathormone, calcitriol, calcium phosphate, cytokines (TNF-α), high-protein, and fat diet increase its expression, whereas estradiol and progesterone decrease [140]. Moreover, in patients with nephrolithiasis urinary OPN values may be decreased; however, results of the studies assessing the role of OPN in renal stone formation are inconclusive [140]. Finally, OPN may be considered as diagnostic and prognostic biomarker of AKI as well as biomolecule with potential for differential diagnosis in patients with cirrhosis; however, further studies in this subgroup may be crucial to define appropriate cutoffs and potential interfering factors, which may be associated with liver dysfunction.

Monocyte chemoattractant protein-1 (MCP-1) is a member of chemokine family [144]. The main role of MCP-1 is to modulate immune response by regulating inflammatory cell trafficking and leukocyte recruitment [144]. MCP-1 is secreted during inflammatory reaction in response to cytokines and seems to be an important protein in the pathogenesis of inflammatory renal diseases [144]. Some studies have shown that urinary MCP-1 levels can be used to diagnose, assess the prognosis and treatment response of various inflammatory and non-inflammatory kidney diseases such as: IgA nephropathy, diabetic nephropathy, or polycystic kidney disease [145]. Similarly to OPN, MCP-1 in patients with cirrhosis enables the AKI diagnosis, due to significant differences between urinary MCP-1 levels in patients presenting with or without AKI. Moreover, MCP-1 values vary between different (1–3) stages of AKI and may be useful in differential diagnosis of acute kidney dysfunction with coexisting liver failure [131]. Relatively low urinary MCP-1 values, yet still higher than in non-AKI patients, have been observed among patients with prerenal AKI, then moderately increased in patients with HRS-AKI and the highest values were associated with ATN-AKI [131].

Shah et al. [70] has shown that renal expression of Toll-like receptor 4 (TLR4) and its urinary excretion was significantly higher among patients with acute deterioration of cirrhosis and accompanying renal dysfunction. TLR4 has confirmed role in HRS pathogenesis, its activation leads to subsequent production of inflammatory mediators. TLR4 was upregulated in renal tubules, mainly in proximal tubule epithelial cells that were subsequently associated with increased values of urinary TLR4 [70]. TLR4 upregulation can be explained by increased dislocation of gut microbiota with subsequent inflammation and TLR4 activation [146]. Upregulation of this receptor corresponds with tubular injury and decreased renal function [146]. Interestingly, increased levels of urinary TLR4 may be revealed due to kidney dysfunction before the sCr creatinine elevation [70]. Moreover, it should be noted that in some patients without renal dysfunction increased levels of urinary TLR4 were also observed, suggesting that urinary excretion of TLR4 may be associated with systemic inflammation [70]. Among patients with systemic inflammatory response syndrome (SIRS), urinary TLR4 was higher in patients with co-occurring kidney dysfunction [70]. To sum up, urinary TLR4 excretion may be modified by systemic inflammation and renal dysfunction [70]. Patients with acute deterioration of liver cirrhosis and non-HRS renal dysfunction and inflammation with coexisting tubular damage have higher values of urinary TLR4, which suggests a potential role of TLR4 in renal injury pathophysiology and indicates the potential role of ATN-AKI as a biomarker [70].

β2-microglobulin (B2M) is a protein present in all cells expressing major histocompatibility complex I (MHC I) that has an important role in onset of immune reaction, particularly in the process of lymphocyte activation [133]. B2M expression is often increased while immune system is stimulated, principally during infections, autoimmune diseases, or certain neoplasms [133]. Serum B2M is filtered in glomerulus with further complete reabsorption in proximal tubular cells, therefore tubular cell damage subsequently causes the increase in urinary B2M levels [133]. It should be noted that isolated glomerular injury can cause the increased urinary B2M, as well as isolated tubular damage; however, urinary B2M levels tend to be higher in patients with ATN [131,133]. Urinary B2M levels, among cirrhotic patients vary depending on the type of AKI [131]. The highest values of urinary B2M have been observed in patients with ongoing ATN-AKI, whereas patients with prerenal AKI or HRS-AKI tended to have lower B2M levels even when compared with cirrhotic patients without AKI [131].

Urinary albumin considered as glomerular injury biomarker often used in CKD categorization and prognosis assessment varied depending on different types of AKI [105,131,132]. Albumin is a high molecular weight protein that, in normal conditions, should not be detected in quantities greater than 30 mg/g creatinine [133]. Most often, albumin is present in the urine in patients with glomerular injury due to the leakage through damaged membrane; however, the greatest values of urinary albumin have been observed among patients with coexisting glomerular and tubular injury [133]. In patients with ATN, urinary albumin levels were the highest, whereas lower levels of urinary albumin were observed in patients with HRS-AKI [132,133]. Therefore, urinary albumin excretion should be considered as a ATN biomarker and may be useful to distinguish between ATN-AKI and HRS-AKI.

#### 3.1.3. Biomarkers of Cell Cycle Arrest

Biomarkers of cell cycle arrest regulating cellular injury repair may have the potential in differentiating between AKI types and assessing the prognosis in patients with kidney dysfunction. To date, there is a little evidence on the potential utility of these biomarkers in AKI diagnosis among patients with cirrhosis [103] Two cell cycle biomarkers are claimed to have a role in clinical outcomes prediction [103]. Insulin-like growth factor binding protein (IGFBP7) and tissue inhibitor of metalloproteinases-2 (TIMP-2) were evaluated in Zhang et al. [147] study, which has shown that these two biomarkers are not able to distinguish between HRS and preserved kidney function in patients with cirrhosis. However, there is evidence for TIMP-2 and IGFBP7 in mortality prediction. It is claimed that increased urinary TIMP-2 and IGFBP7 values can indicate ongoing reversible renal tubular stress damage; however, there were no associations between HRS-AKI occurrence and values of these biomarkers observed [147]. Further studies are crucial to determine the role of cell cycle arrest biomarkers in patients with cirrhosis and decreased renal function.

### 3.2. HRS Diagnostic Criteria

In 2015, the ICA published a revised consensus introducing new recommendations on HRS diagnosis due to several limitations of the diagnostic criteria used so far [104]. General changes implemented by the consensus were mainly related to new definition and diagnostic criteria of AKI, proposed and validated in patients without cirrhosis [104,105]. Recent KDIGO guidelines define AKI as any of the following criteria: (1) increase in sCr by ≥ 0.3 mg/dL; (2) increase in serum creatinine ≥ 1.5 times baseline, which is known or presumed to have occurred within the prior 7 days; or (3) urine volume < 0.5 mL/kg/h for 6 h [105]. Due to the different pathophysiology of acute kidney dysfunction in patients with liver cirrhosis, the use of new AKI diagnostic criteria in patients with liver disease required evaluation [104]. Moreover, the use of reduced urine output in patients with cirrhosis and/or ascites may be improper, due to the tubular sodium reabsorption, subsequent glomerular hypofiltration leading to sodium retention and oliguria [148,149]. Therefore, patients suffering from ascites are often treated with diuretics that cause an increase in urine output and misleads renal function assessment [104]. Thus, ICA proposed a definition of acute kidney dysfunction in patients with cirrhosis—HRS-AKI, a renewed definition of HRS type 1 [104]. Both AKI and HRS-AKI diagnostic criteria are presented and compared in Table 3 [104,105].

Notwithstanding, many patients do not meet criteria for HRS-AKI; however, in some of these patients HRS diagnosis will be appropriate. In patients with chronic renal dysfunction and liver insufficiency, the diagnosis of HRS-NAKI (non-AKI) should be considered. HRS-NAKI is a kidney injury not meeting the criteria of HRS-AKI and can be subdivided into two subgroups: HRS-chronic kidney disease (HRS-CKD) and HRS-acute kidney disease (HRS-AKD) [18]. HRS-CKD and HRS-AKD are defined as decreased eGFR < 60 mL/min/1.73 m^2^ for at least 3 months or less than 3 months, respectively [18]. Both CKD and HRS-CKD diagnostic criteria are presented and compared in Table 4.

### 3.3. Differential Diagnosis

To diagnose HRS-AKI or HRS-NAKI, all structural renal injuries should be excluded. Therefore, renal imaging, especially ultrasound and urine microscopy, can be performed with accompanying urine sodium excretion assessment [18,105]. In recent years, several novel biomarkers to distinguish between different types of AKI have been proposed and evaluated (see Section 4.1. Renal dysfunction biomarkers in patients with impaired liver function). For many of them, available data are highly limited. It is noteworthy that various types of renal injury can develop in patients with liver cirrhosis, mainly prerenal AKI (46–66% of all cases), HRS-AKI, acute tubular necrosis AKI (ATN-AKI), and postrenal AKI [18,122].

#### 3.3.1. Prerenal AKI and HRS-AKI

Prerenal AKI occurs in approximately 46–66% of acute renal dysfunction cases among patients with cirrhosis. It can be effectively managed and reversed by serum volume expansion that contemporaneously exclude HRS-AKI. Patients with cirrhosis are more susceptible to prerenal AKI due to the use of diuretics, gastrointestinal fluid loses caused by laxatives in prevention of liver encephalopathy, and large volume paracentesis without albumin supplementation [18,122]. There are several clinical conditions leading to decreased renal function and predisposing to prerenal AKI, e. g., heart failure causing decreased CO and renal hypoperfusion due to RAAS and sympathetic compensatory activation [105,122]. Furthermore, medication that the patient is taking should be considered in the assessment and differential diagnosis of patient with suspected AKI. There are several drugs leading to decreased serum volume or renal hypoperfusion. Apart from the already mentioned diuretics and laxatives, it should be noted that, in certain clinical states, especially heart failure, several drugs can predispose to prerenal AKI. Commonly used in heart failure, beta-blockers taken in improper doses may aggravate cardiac dysfunction and lead to renal hypoperfusion due to reflex RAAS and sympathetic activation [150,151]. Recent data suggest that sodium-glucose cotransporter 2 inhibitors (SGLT2i), commonly used for DM, HF, and CKD treatment, can cause serum volume depletion with the risk of prerenal AKI; however, it is more specific for SGLT2i to cause urinary tract infections rarely with urosepsis and pyelonephritis [105,151,152]. Recent trials assessing cardiovascular outcomes of SGLT2i report that AKI occurred with similar frequency in both SGLT2i and placebo groups. The mechanism of AKI in SGLT2i therapy remains unknown; however, it is suspected that dehydration caused by osmotic diuresis may predispose to prerenal AKI [152]. Therefore, in the diagnostic process of AKI the effect of beta-blockers, SGLT2i and other drugs taken by the patient should be considered.

In patients with heart failure and severely decreased renal function, serum volume expansion may cause overhydration, often leading to heart failure decompensation [150,151]. According to ICA consensus, there is no parameter to assess the volume status and safety of volume expansion, while a two-day fluid challenge is recommended [104]. A pilot observational study that was conducted to evaluate the utility of ultrasonographic assessment of inferior vena cava diameter (IVCD) and collapsibility index (IVCCI) in an assessment of the volume status in patients presenting with cirrhosis and signs of AKI [92]. The results of the study have shown that only 28% of patients with suspected HRS-AKI were fluid depleted, 8% had intraabdominal hypertension (IAH), and 11% of patients were fluid expanded [92]. Ultrasonographic assessment confirmed euvolemia only in 36% of patients [92]. In all fluid-depleted patients, intravenous (i.v.) albumin has been administered, fluid-expanded patients were treated with loop diuretics, and IAH patients had large volume paracentesis [92]. In 30% of overhydrated patients treated with diuretics, the significant improvement of renal function have been observed [92]. Results of the study suggest that only 28% of patients presenting with AKI had fluid depletion potentially leading to prerenal AKI [92]. Moreover, initial ultrasonographic classification precipitate the treatment onset and increase the safety of diagnostic process especially in patients with high risk of fluid retention [92,150].

Schrezenmeier et al. [123] reviewed useful biomarkers in HRS diagnosis and proposed that the suspicion of prerenal AKI can be verified by comparing NGAL and sCr concentrations. Normal NGAL values with a simultaneous increase in sCr may indicate the absence of renal stress damage; however, functional impairment may be present due to prerenal-AKI [123]. Urinary NGAL concentrations in prerenal AKI are similar to patients with normal kidney function or stable CKD [130]. Therefore, in patients presenting signs and symptoms of AKI, normal NGAL values and elevated sCr without signs of overhydration serum volume expansion seems to be the right differential diagnostic approach and treatment. Moreover, Gowda et al. [122] evaluated the utility of fractional sodium and urea excretion (FENa and FEUrea) to distinguish between HRS-AKI and prerenal-AKI. The results of the study indicate that FENa as well as FEUrea cannot distinguish between prerenal AKI and HRS-AKI due to the low sensitivity and specificity [122].

#### 3.3.2. ATN-AKI and HRS-AKI

In contradistinction to prerenal AKI, ATN-AKI does not respond to plasma volume expansion and often require renal replacement therapy [104,105]. Due to the different management methods among AKI types, the initial diagnosis should be made early and proper treatment should be instituted. Already described biomarkers (see Section 3.1.2) such as NGAL, IL18, L-FABP, and KIM-1 seem to have a great potential for differentiating between HRS-AKI and ATN-AKI [122,123,131]. It should be noted that currently available data are limited and further studies to establish the role and define cutoffs for these biomarkers are needed. Increased values of ATN biomarkers suggest ongoing tubular injury with following acute renal dysfunction, whereas HRS-AKI is less possible. Single studies assessed the potential role of other novel biomarkers including TFF-3, GST, OPN, MCP-1, and TLR4 [70,131,146]; however, due to the lack of data, further evaluation of their utility in differential diagnosis of acute renal dysfunction among patients with cirrhosis. Moreover, several studies suggest the ability to distinguish between ATN-AKI and HRS-AKI by measuring FENa and FEUrea [122,153]. Results of these studies indicate that increased values of FENa and FEUrea suggest ATN-AKI occurrence [122]. These two values tend to be low in patients with preserved tubular function, whereas FENa and FEUrea may be increased secondary to tubular injury, causing decreased sodium and urea reabsorption [122,153]. Several publications suggest that in patients with FENa > 1%, diagnosis of HRS-AKI should be avoided [153]. It should be noted that FENa values vary depending on the liver function, where severe cirrhosis may cause renal hypoperfusion with secondary decreased sodium filtration in glomerulus [122]. Finally, FENa seems to have better specificity and sensitivity in differentiating ATN-AKI from HRS-AKI when compared to FEUrea; however, it is reported that novel biomarkers, especially NGAL seem to be most accurate differential biomarker [131,154].

To sum up, increased ATN biomarkers suggest ATN-AKI rather than HRS-AKI occurrence. Moreover, increased urinary FENa, combined with increased novel biomarkers may indicate ongoing tubular injury that is more probable cause of AKI than HRS. Further studies are needed to establish the cutoff values of these biomarkers and evaluate their specificity and sensitivity.

#### 3.3.3. Postrenal AKI and HRS-AKI

Postrenal AKI is most often caused by inability to eliminate urine from the kidney through the renal collecting system due to obstructed urinary flow [155]. Extrarenal obstruction such as prostatic hyperplasia, neurogenic bladder, retroperitoneal fibrosis, or urinary tract neoplasms may be the cause of postrenal AKI [156]. The most important historical findings that suggest postrenal AKI are urinary urgency (in prostatic hyperplasia), polyuria, renal and urinary stones, urinary tract neoplasms, and gross hematuria [156]. Renal ultrasound should be performed in all patients presenting with AKI to exclude potential obstruction causing postrenal AKI [105,156]. Moreover, pelvic and abdominal computer tomography or magnetic resonance scan may be considered to reveal other causes of obstruction, such as pelvic tumors or retroperitoneal fibrosis [156]. There is no biomarker with enough high specificity and sensitivity to diagnose postrenal AKI. The diagnosis should be made based on signs, symptoms, and diagnostic imaging [105,156].

## 4. Treatment of HRS-AKI

### 4.1. Supportive Management

As the prognosis for HRS-AKI is rather poor, treatment should be administered as soon as a diagnosis is made so that further deterioration of kidney function can be prevented [157]. Moreover, an attentive search for any possible reversible causes such as hypovolemia, drug-induced nephrotoxicity, or urinary tract obstruction should be conducted [158]. Due to the risk of severe, especially cardiovascular complications, all of the patients with HRS-AKI should have parameters such as standard vital signs, liver, and renal tests, urine output, fluid balance, and arterial pressure closely monitored [11,157]. Additionally, intravascular volume status monitoring (via measurement of the central venous pressure or assessment of the inferior vena cava) can be helpful in managing fluid balance and preventing volume overload and diluted hyponatremia [11,157]. Moreover, urine volume monitoring is also particularly important, as oliguria has been associated with poorer outcomes [158,159]. Patients should be carefully screened for possible complications, such as a deterioration in kidney function and bacterial infections in particular, as there is no data supporting the administering of antibiotics as prophylactic treatment for an unproven infection [59,158,160]

Closely after the diagnosis of HRS-AKI, it is advised to immediately withdraw all nephrotoxic drugs, such as vasodilators or non-steroidal anti-inflammatory drugs, and all diuretics [11,104,158,161]. Spironolactone is especially contraindicated, due to the risk of severe hyperkalemia [11]. Although there are some controversial data, it is advised to withhold beta-blockers as well [158,161,162,163].

### 4.2. Pharmacological Therapy

The revision of the diagnostic criteria for HRS-AKI allows for a diagnosis at an earlier stage and a quicker initiation of treatment, as it is speculated that patients are now able to receive treatment approximately 4 days earlier [164]. This change may likely result in overall better treatment outcomes because lower serum creatinine levels before the administration of pharmacotherapy may correspond to a transient course of AKI [165]. However, it is crucial to note that many published studies use the old classification and terminology and refer to HRS-AKI as type-1 HRS, and therefore, it is unknown whether these results apply to patients that do not fit the criteria for type-1 HRS diagnosis [43,158,161].

Both the European Association for the Study of the Liver (EASL) and the American Association for the Study of Liver Diseases (AASLD) recommend vasoconstrictors in combination with albumin as the mainstay of HRS-AKI treatment [158,161]. It is noteworthy that the efficacy of the treatment should be monitored carefully. In relation to that, EASL recommends treatment efficacy assessment and defines a complete response to the treatment as serum creatinine within 0.3 mg/dL (26.5 µmol/L) from the baseline value and a partial response as a regression of AKI stage to a final serum creatinine of ≥0.3 mg/dL (26.5 µmol/L) from the baseline value [161].

Treatment options comprising well-established therapies as well as novel management methods are presented in Table 5.

#### 4.2.1. Vasoconstrictive Drugs

Vasoconstrictors act by opposing splanchnic vasodilation, thereby improving renal blood flow [28,161,166]. Renal perfusion corresponds with changes in MAP, and an increase in MAP caused by implementing a vasoconstrictor is associated with a higher chance of reversing HRS [18,167]. Vasoconstrictive therapy should be continued until creatine returns to baseline levels, usually up to 14 days, although some patients may require prolonged treatment [158]. However, therapy may be discontinued if creatinine levels remain at or above pretreatment levels over 4 days with the maximum tolerated doses of the drugs [158]. There are three classes of vasoconstrictors with established efficacy in HRS-AKI treatment: vasopressin receptor agonists (such as vasopressin, terlipressin, ornipressin), alpha-adrenergic receptor agonists (like noradrenaline and midodrine) and somatostatin receptor agonists (namely octreotide) [11].

##### Terlipressin

Terlipressin in combination with albumin is recommended as a first choice of treatment by the EASL [161]. It activates the vasopressin 1A receptor of the vascular smooth muscle cells, subsequently inducing vasoconstriction [168]. It also acts through vasopressin 1B receptors to increase levels of adrenocorticotropic hormone and cortisol and therefore may oppose the relative adrenal insufficiency, which is common in patients with decompensated cirrhosis [18,169]. Terlipressin exerts a greater affinity for the vascular V1A receptor than for the renal V2 receptor [19,168]. Several studies have proved the efficacy of terlipressin and albumin therapy with the response rate ranging from 64 to 76% [161,170,171,172]. Moreover, terlipressin seems to be a very suitable treatment option for patients with systemic inflammatory response, alcohol-associated hepatitis, and sepsis, as studies have shown that their response may be better than in patients without these factors [18,21,170,173,174,175]. The EASL advises administering terlipressin as i.v. boluses at an initial dose of 1 mg every 4 to 6 h [161]. However, continuous i.v. infusion of terlipressin at an initial dose of 2 mg/day may be more beneficial for some patients as it allows for a reduction in the global daily dose of the drug and therefore the rate of its adverse effects [161,172]. The most common side effects of terlipressin include diarrhea, abdominal pain, circulatory overload, and cardiovascular ischaemic complications [161].

##### Noradrenaline

Noradrenaline is a systemic vasoconstrictor that acts via the α_1_-adrenergic receptor in the vascular smooth muscle cells, and, as such, it increases peripheral vascular resistance and thus the MAP, which results in an improvement in renal perfusion [164]. While noradrenaline in continuous i.v. infusion at a dose of 0.5–3 mg/h seems to be as effective as terlipressin in treating HRS-AKI, there is not enough data for it to be a conclusive claim [19,161,176,177,178]. Moreover, a recent study by Arora et al. [179] has shown that noradrenaline is inferior to terlipressin in reversing HRS-AKI and improving the overall survival rate. However, a meta-analysis from 2017 suggests that noradrenaline therapy should be favored over the midodrine–octreotide combination [19,179]. The adverse effects of noradrenaline include ischemic complications, cardiac arrhythmias, and respiratory side effects [174].

##### Midodrine and Octreotide

Midodrine, like noradrenaline, is a systemic vasoconstrictor that acts via activation of α_1_-adrenergic receptors on vascular smooth muscle cells [18]. Octreotid is a somatostatin analog, a splanchnic vasodilator, and a direct mesenteric vasoconstrictor [18,180]. Both midodrine and octreotide alone in monotherapies are unable to improve renal function in patients with HRS-AKI; however, a combination of midodrine and octreotide, while having a slow effect, seems to be able to reverse HRS [18,164,180,181,182]. Nonetheless, midodrine and octreotide combination therapy is significantly less effective than terlipressin [171].

#### 4.2.2. Albumin

Albumin, the main blood plasma protein that maintains colloid osmotic pressure and thus intravascular volume, is decreased in patients with cirrhosis [183]. Studies have shown that albumin exerts many beneficial qualities, such as volume expansion, a positive cardiac inotropic effect, antioxidant, and immunomodulatory properties, therefore its infusion is essential for the appropriate management of HRS-AKI [15,184,185,186,187]. It is also suggested that albumin may reduce endothelial activation [188]. While albumin alone is ineffective in treating HRS-AKI, studies have shown that terlipressin used alone is less effective than terlipressin in combination with albumin [175,189,190,191]. The appropriate dose of albumin in HRS-AKI therapy has not been well established [161]. The EASL suggests administering 20% albumin solution at a dose of 20–40 g/day, optimizing the dose by assessing central blood pressure to prevent circulatory overload [161].

### 4.3. Transjugular Intrahepatic Portosystemic Shunts (TIPS)

As portal hypertension plays a role in the development of HRS-AKI, the creation of an intrahepatic shunt should theoretically be beneficial in improving renal function [27]. Only a few studies have assessed the effectiveness of TIPS in patients with HRS; however, there are some data suggesting that the creation of TIPS may lead to an improvement in kidney function, a reduction in plasma renin activity, aldosterone, and norepinephrine concentrations, as well as an improvement in serum creatinine, serum sodium, and urine output [192,193,194,195]. Nonetheless, there is not enough evidence to support recommending TIPS as a standard treatment for HRS-AKI [158,161,196,197]. Moreover, the clinical applicability of TIPS may be limited since it is contraindicated in many patients with HRS-AKI, because of their severe level of liver failure [161]. However, it is suggested that TIPS may exert a protective effect against the development of HRS-AKI in patients with cirrhosis [198].

### 4.4. Renal Replacement Therapy (RRT)

Initiation of RRT in HRS-AKI is controversial and is typically viewed as a bridge to transplantation in listed patients [27,164]. The EASL suggests that it should be considered in patients unresponsive to vasoconstrictors and those with end-stage kidney disease [161]. The indications for RRT are the same in patients with cirrhosis as in the general population, the decision to start RRT should be made on clinical grounds, including increasing volume overload, symptomatic azotaemia, worsening kidney function, electrolyte disturbances such as severe acidosis, hyponatremia, or hyperkalemia not improving with medical management and diuretic intolerance [161,165]. However, data have shown that RRT does not improve the survival rate or kidney function of patients with HRS-AKI and therefore RRT itself should not be viewed as a treatment [11,199,200]. RRT can be beneficial in patients awaiting liver transplant as it can optimize the electrolyte balance and volume status prior to the procedure, while the decision to administer RRT should be based on the individual severity of illness in those who are not candidates for a transplant to avoid futility [11,26,161]. However, it should be noted that a recent study by Staufer et al. [201] has found that mortality among patients with critical-stage cirrhosis and a need for RRT is substantially high, independent of LT options. Therefore, the EASL suggests repeated risk stratification during the procedure with the assistance of prognostic scores, clinical judgment, and patients’ preferences [161,201]. There are no data available regarding the most beneficial time to start RRT, although it is suggested that early RRT might improve survival [161,202,203,204]. Utako et al. [205] in a meta-analysis confirmed that prolonged RRT is associated with a negative impact on post-transplant patient and renal outcomes. While both intermittent and continuous renal replacement therapy (CRRT) can be used in patients with HRS-AKI, CRRT has been suggested to be a more preferable option as it provides greater cardiovascular stability and allows for a slower correction of severe or refractory hyponatremia than hemodialysis [161,164].

### 4.5. Liver Transplant (LT)

A liver transplant is the best treatment for HRS-AKI as it cures the underlying causes of the disease, which are mostly portal hypertension and liver dysfunction [161,164]. However, it is important to note that the presence of HRS at the time of LT has a negative impact on the survival rate after the procedure [161,206]. It is advised for patients with HRS-AKI to be referred for liver transplant assessment as soon as possible [164]. Successful LT restores hepatic function, reduces serum aldosterone and renin levels, improves systemic blood pressure, normalizes renal resistive indices, and increases renal sodium excretion [11,207]. Patients awaiting LT are prioritized due to an increase in sCr and an associated rise in the Model for End-Stage Liver Disease (MELD) score [164]. As vasoconstrictive therapy lowers the sCr value and the MELD score, and thus reduces the priority for LT, there has been some controversy as to whether patients with HRS-AKI should be treated as some fear pharmacotherapy might delay the transplant [161,164,174]. Nonetheless, medical treatment should not be withheld, as the benefits of effective management of the disease outweigh the potentially dangerous consequences of prolonging the wait for LT [174]. Moreover, a study by Piano et al. [208] has shown that terlipressin in combination with albumin can be associated with a reduction in the requirement for LT itself, an improvement in 30 day pretransplantation survival, a reduction in the risk of chronic renal disease after LT, and a need for RRT after LT. However, a study by Boyer et al. [207] has found that terlipressin in combination with albumin had no significant impact on posttransplant survival. Despite the inconclusive data, both the EASL and the AASLD indicate that patients with HRS should be treated prior to transplantation as it may improve their outcomes after LT [158,161]. General indicationsfor liver transplant with MELD score are summarized in Table 6.

### 4.6. Simultaneous Liver-Kidney Transplantation (SLKT)

While LT restores kidney function, it is difficult to predict the scale of the improvement [27,174]. The presence of AKI before LT has been associated with an increased risk of CKD and a higher mortality rate after the transplant [211,212]. Data suggest that around 25% of patients remain in need of dialysis after LT [213,214]. Therefore, SLKT can be considered a treatment option for HRS-AKI patients with a questionable chance for a renal recovery with a liver transplant alone [164]. However, the survival benefit of SLKT over LTA alone is unclear [164]. A study by Sharma et al. [215] found that the survival advantage of SLKT over LTA was of marginal clinical significance among patients who were not on dialysis and were present only with a donor kidney of sufficient quality. While the SLKT in patients with HRS-AKI remains controversial, the EASL states that it should be considered in patients with significant CKD or with sustained AKI, including HRS-AKI, with no response to drug therapy [161]. The indications for simultaneous liver-kidney transplantation are presented in Table 7.

### 4.7. Novel Therapies

#### 4.7.1. Molecular Absorbent Recirculating System (MARS)

MARS is an extracorporeal artificial liver support system that circulates albumin in order to remove cytokines and bacterial products and thus combat vasodilation [11,14,66]. The system is approved as a form of treatment for hepatic encephalopathy [11]. MARS is able to remove molecules that accumulate with liver and kidney failure, such as bilirubin, ammonium, urea, creatinine, inflammatory cytokines, and vasoactive mediators, and thus potentially restore hemodynamic derangements that occur in HRS [11,216,217]. A trail by Mitzner et al. [218] has shown a significant reduction in serum creatinine and mortality in patients treated with MARS, standard medical treatment, and hemodiafiltration, compared to those who were treated with just standard medical treatment and hemodiafiltration. However, the RELIEF trial [219] has failed to find any statistically significant reduction in mortality rates in patients treated with MARS compared to standard medical therapy. A study by Wong et al. [220] has shown that MARS was not effective in improving systemic hemodynamics and renal function in patients with HRS not responding to vasoconstrictor treatment. Therefore, MARS’s place in the management of HRS-AKI is currently uncertain, the EASL has not yet recommended MARS for HRS treatment but has suggested that a further investigation into the potential benefits of this form of treatment is needed [14,161].

#### 4.7.2. Fractionated Plasma Separation and Adsorption System (Prometheus)

Prometheus is an extracorporeal artificial liver support system device that removes albumin-bound and water-soluble toxins using the method of fractionated plasma separation and adsorption (FPSA), it is similar to MARS but uses a different membrane and the patient’s own albumin rather than an exogenous one [221,222,223]. There are some data suggesting FPSA to be a more effective detoxification method than MARS [224]. A study by Rifai et al. [224] has found Prometheus to be safe to use in patients with HRS. Another study by Rifai et al. [223] has shown Prometheus to be more effective in reducing ratios of bilirubin, ammonia, and urea. However, a study by Kribben et al. [225] has found that extracorporeal liver support with FPSA does not result in a survival benefit. Thus, since the data are still inconclusive, the EASL does not recommend the FPSA method to be used in HRS-AKI patients but suggests a further investigation into its benefits [161].

#### 4.7.3. Therapeutic Plasma Exchange (TPE)

TPE, also known as plasmapheresis, uses an extracorporeal apheresis device to separate and remove plasma from whole blood and then return the cellular blood components with replacement fluid to a patient’s body [226]. Thus, it allows liver function to recover through the reduction of systemic inflammatory mediators and the removal of toxic substances [164]. This form of treatment has been shown to be beneficial in acute liver failure and acute on chronic liver failure [227,228]. However, none of these studies have evaluated an improvement of renal function specifically in patients with HRS-AKI, so it is unknown whether it is actually beneficial [164].

#### 4.7.4. Plasma Diafiltration (PDF)

PDF is a blood purification procedure that combines dialysis with plasma filtration and has been used to treat acute liver failure as well as other conditions [229]. PDF has certain advantages over systems like MARS or Prometheus, as it is less expensive and does not involve as much preparation [230]. A case report by Nakae et al. [230] has shown that administering PDF helped a patient recover from HRS and significantly reduced his levels of total bilirubin, interleukin-18, creatinine, and cystatin C. However, there is not enough evidence to support recommending a standard use of PDF in HRS-AKI and more studies are needed [230].

#### 4.7.5. Selepressin

Selepressin is a novel selective vasopressin 1A receptor agonist that has recently been examined as an alternative to vasopressin in the treatment of septic shock [18,231]. Unlike terlipressin, which has a low affinity for renal vasopressin 2 receptors, selepressin poses a lower risk of unwanted solute-free water absorption and aggravating hyponatremia and volume overload [18,168,232]. The high selectivity of selepressin towards V1A receptors makes it a promising agent as a potential treatment option for HRS-AKI; however, more data are needed [18].

#### 4.7.6. Serelaxin

Serelaxin (a recombinant human relaxin-2) is a peptide molecule with anti-fibrotic and vasoprotective properties that binds to relaxin family peptide receptor-1 (RXFP1) and has been shown to improve renal perfusion [233]. A trial by Snowdon et al. [233], which studied the effect of serelaxin on cirrhotic rats, has shown that it was able to improve renal perfusion by reducing renal vascular resistance. Serelaxin was compared to terlipressin in cirrhotic patients with renal impairment, and it was found that serelaxin improved the total renal arterial blood flow, was safe and well tolerated, whereas terlipressin did not cause any significant change in renal arterial blood [233]. However, it has not been tested on patients with HRS-AKI yet [11].

#### 4.7.7. Nebivolol

Nebivolol is a nonselective vasodilator beta-blocker that has been studied in rats with an induced HRS and was found to have antioxidant, anti-inflammatory, and antiapoptotic properties with renoprotective and hepatoprotective effects [234]. These results place nebivolol as a potential add-on or preventative drug for patients with HRS-AKI, although more studies are needed [235].

#### 4.7.8. Pentoxifylline

Pentoxifylline is a phosphodiesterase inhibitor with anti-TNF-α activity, which can potentially reduce inflammation by decreasing proinflammatory cytokines [11,235]. A trial by Da Silva et al. [236] has found that adding pentoxifylline to albumin with midodrine and octreotide was safe in patients with HRS and had the potential to be used as a new treatment strategy. Another trial by Akriviadis et al. [237] has found that pentoxifylline significantly decreased the risk of developing HRS and improved short-term survival in patients with severe alcoholic hepatitis. Yet another study by Lebrec et al. [238] has found that, while pentoxifylline did not reduce short-term mortality in patients with advanced cirrhosis, it decreased the risk of complications. However, as of now, more studies are needed to confirm these results, and currently the EASL does not recommend the standard use of pentoxifylline as a preventative treatment [11,161].

#### 4.7.9. Rifaximin

Rifaximin is a poorly absorbed, gut-selective broad-spectrum antibiotic [239]. It is suggested that it may reduce small intestinal bacterial overgrowth in cirrhotic patients, which is associated with systemic endotoxemia and can lead to worsening of portal hypertension [240,241]. Studies have shown that rifaximin was able to significantly reduce the overall blood urea nitrogen and serum creatinine concentrations in patients with cirrhosis and ascites compared to the group who received standard medical care and thus reduce the risk of HRS-AKI [235,239]. A study by Kang et al. [242] has shown that rifaximin was able to reduce levels of interleukin 6, tumor necrosis factor-α (TNF-α), and endotoxins. Studies by Kamal et al. [243] and Dong et al. [244] have shown that rifaximin was able to decrease the risk of developing HRS-AKI in cirrhotic patients, making it a suitable preventative treatment. However, a study by Kang et al. [240] has not confirmed rifaximin to reduce the risk of HRS-AKI, possibly because of its multifactorial pathophysiology, except for endotoxemia and therefore, as of now, the data are inconclusive.

#### 4.7.10. Fucoidan

Fucoidan, which comprises a wide range of fucans, has been shown to possess multiple beneficial qualities, such as antiviral, antioxidant, antitumor, anti-inflammatory, and anticoagulant [245,246,247,248,249,250]. It has been found to have a protective effect on liver injury as well as the potential to alleviate renal injury in mice [251,252]. A recent study by Zhao et al. [245] has found that administering fucoidan in mice with bile duct ligature-induced HRS alleviated HRS via inhibition of the renal Ostα and Ostβ, as well as reduced bile acids reabsorption and significantly decreased levels of alanine aminotransferase and aspartate aminotransferase, uric acid, creatinine, and uric nitrogen. These results pose fucoidan as a potential therapeutic strategy for HRS, although more studies are needed [245].

#### 4.7.11. Stem Cell Therapy

It is speculated that mobilization of bone marrow-derived stem cells with granulocyte colony-stimulating factor (G-CSF) could promote hepatic regeneration [253]. Studies by Garg et al. [253] and Saha et al. [254] have assessed the impact of G-CSF on patients with ACLF and have found that it prevented the development of HRS. However, a recent study by Colli et al. [255] has shown that, while G-CSF therapy seemed to decrease mortality in patients with decompensated advanced chronic liver disease, it had no significant effect on the development of HRS, thus, since the evidence is uncertain, it is unknown whether stem cell therapy can be safely used to prevent HRS-AKI.

### 4.8. Prevention of HRS-AKI

It is crucial to diagnose HRS as early as possible and to exclude possible other causes of renal failure in patients with cirrhosis such as hypovolemia, shock, renal parenchymal disease, or the use of nephrotoxic drugs.

There is evidence that administration of albumin can potentially play a protective role against HRS, the EASL recommends administering albumin at a dosage of 1.5 g/kg at diagnosis and then increasing the dosage to 1 g/kg on day three in order to prevent AKI in patients with SBP [161]. Studies have found this therapy to be effective in reducing the risk of HRS and the mortality rate in patients with SBP [56,256]. The EASL also recommends the administration of norfloxacin at a dosage of 400 mg/day as prophylaxis with norfloxacin has been found to reduce the incidence of spontaneous bacterial peritonitis, delay the development of HRS, and improve overall survival in patients with advanced cirrhosis [161,257].

Gut microbiota translocation among patients with cirrhosis increases the risk of spontaneous bacterial peritonitis (SBP) that may increase the mortality [161]. The risk of SBP in cirrhotic individuals presenting with HRS may be decreased with antibiotic prophylaxis and careful monitoring for the signs of peritoneal infection [161]. EASL recommends primary antibiotic prophylaxis of SBP in patients with Child-Pugh score ≥ 9 (Table 8) and serum bilirubin ≥ 3 mg/dL with impaired renal function or hyponatremia and ascitic fluid protein < 15 g/L [161,258]. The currently recommended option primary prevention of SBP in patients with cirrhosis, as well as in patients recovering from SBP is orally administered norfloxacin (400 mg/d) [161]. Due to higher complexity, treatment of SBP in acute phase is outside the scope of this article.

Decreased intravascular volume is one of the mechanisms for the development of HRS in patients with cirrhosis. It is therefore recommended that all diuretics should be discontinued during the initial assessment and diagnosis of HRS [161]. It is intended to prevent further decline in EABV. There have been studies that have evaluated the effect of furosemide in combination with dopamine and albumin and these have shown a similar effect on improving urine output and urinary sodium excretion as terlipressin with albumin [259,260]. However, this requires further research, and the use of any diuretics is not currently recommended. Furthermore, it is recommended to withdraw vasodilators, non-steroidal anti-inflammatory drugs, or beta-blockers, which would reduce CO through their negative inotropic effect [15,161]. It is important to carefully monitor patients with HRS in terms of urine output, fluid balance, and blood pressure so as not to dehydrate them, but also to prevent volume overload [161].

## 5. Conclusions

HRS is a form of kidney function impairment that characteristically occurs in cirrhosis. Significant improvements have been achieved in the diagnosis and management of HRS in recent years. The pathogenesis of HRS is intricate and results from the interaction of numerous pathophysiological mechanisms in cirrhotic patients. The most important are considered to be circulatory disorders resulting from hypertension and progressive cirrhotic cardiomyopathy. The effect of these disorders is the activation of RAAS, SNS, and the release of vasopressin to maintain EABV; however, they contribute to reduced renal blood flow. Other mechanisms with a key role in the development of HRS is a persistent elevated inflammatory response despite the absence of overt infection, which is associated with the presence of PAMPs and DAMPs. These molecules contribute to the increased expression of TLR4 and caspase 3 in renal tubular cells, leading to the development of acute renal dysfunction through cell damage and apoptosis of these cells. In addition, RAI, cholestatic nephropathy, chronically elevated intra-abdominal pressure, or the direct neuronal connection between the liver and kidneys have been shown to be involved in the development of HRS.

Although many studies have been conducted in recent years, the diagnosis and treatment of HRS and its subtypes can be difficult. One of the greatest challenges in the diagnostic process of renal dysfunction in patients with liver dysfunction is to distinguish between different types of AKI. First of all, it should be noted that sCr, being the one of the most important biomarkers assessing renal function, should be considered less reliable and can overestimate renal function in patients with cirrhosis. Therefore, it is assumed that cystatin C would be more accurate functional biomarker to estimate GFR. Another challenge is to differentiate between AKI types that can occur in cirrhotic patients. Despite many studies conducted in recent years searching for a biomarker potentially useful HRS-AKI diagnosis, there is still no easily accessible cost-effective biomarker available. Several biomarkers such as NGAL, KIM-1, L-FABP, or IL-18 are able to exclude the ATN-AKI with great accuracy; however, for some of them the exact specificity and sensitivity remain unclear and emphasize the need for further studies on different populations. Finally, only a few studies explored the utility of TFF-3, B2M, TLR4, OPN, GSTs, and TLR4, whereas the results indicated their potential for differential diagnosis. Therefore, all of these markers should be the subject of future research, and the understanding of their kinetics and interfering factors may be important for future HRS diagnostic methods.

The prognosis for HRS-AKI is rather poor, and thus the treatment should be administered as soon as the diagnosis is made, and patients should be closely monitored for any possible complications. As of now, the recommended treatment strategy is administering terlipressin with albumin, as it is a well-studied and effective combination. Liver therapy is the preferred curative treatment for HRS-AKI; however, it is not available for all patients. Therefore, there is a great need for newer therapies to improve the survival rate of patients with HRS-AKI. New treatment strategies targeting systemic inflammation, DAMPs and PAMPs, downstream signaling, and specific pathophysiological mechanisms of HRS-AKI should be explored.

## Figures and Tables

**Table 1:** Clinical types of HRS, according to Kidney Disease: Improving Global Outcomes (KDIGO) [14].

HRS-AKI	HRS-CKD
Rapid deterioration in renal function with acute kidney injury, marked increase in serum creatinine, typically defined by a significant decrease in kidney function over a short period (usually less than two weeks)	Slower, more gradual decline in kidney function, representing a chronic kidney disease state, often characterized by a chronic, insidious onset in patients with advanced liver disease and associated hepatic and circulatory abnormalities

**Table 2:** Characteristics of sCr and cystatin C as renal functional biomarkers [117,118,119,120,121].

	Creatinine	Cystatin C
Formation	Mostly Skeletal Muscles	All Nucleated Cells
Elimination	Renal Filtrated in glomeruli receptor-mediated tubular secretion Factors influencing tubular handling: Drugs: cimetidine, thrimetoprim, fenofibrate, dolutegavir, ritonavir, tyrosine kinase inhibitors, diuretics, CCB—decrease tubular secretion Low GFR—increase tubular secretion Extrarenal elimination Bacterial creatininase Factors increasing extrarenal elimination: Antibiotics, low GFR	Renal Filtrated in glomeruli Factors influencing tubular handling: Corticosteroids increase glomerular filtration of cystatin C
Clinical conditions modifying serum levels of biomarker	Increase: High muscle mass (body builders) High protein and creatine diet Decrease: Vegetarian and vegan diet Chronic disease Muscle wasting, frailty, cachexia	Increase: High fat mass (obesity) Smoking Hypertension Nephritis Hyperthyroidism Decrease: Hypothyroidism Hypercortisolism

Abbreviations: CCB—calcium canal blockers; GFR—glomerular filtration rate.

**Table 3:** AKI and HRS-AKI diagnostic criteria [18,105].

AKI	HRS-AKI
Meeting one of the following criteria is sufficient for diagnosis: increase in sCr by ≥0.3 mg/dL within 48 h; increase in sCr ≥ 1.5 times baseline, which is known or presumed to have occurred within the prior 7 days; urine volume < 0.5 mL/kg/h for 6 h	increase in sCr by ≥0.3 mg/dL within 48 h * increase in sCr ≥ 1.5 times baseline, sCr value within previous 3 month can be considered as baseline * no response to diuretic withdrawal and 2-day fluid challenge (20–25% albumin, 1 g/kg/day) Absence of shock Cirrhosis with ascites Excluded recent use of nephrotoxic drugs No signs of structural renal injury Normal renal ultrasound Absence of proteinuria Absence of hematuria

* There is no minimum sCr value allowing the diagnosis of HRS-AKI. AKI—acute kidney injury; HRS-AKI—hepatorenal syndrome acute kindey injury; sCr—serum creatinine.

**Table 4:** CKD and HRS-CKD diagnostic criteria [18,105].

CKD	HRS-CKD
Either of the following for at least 3 months One or more Albuminuria (AER ≥ 30 mg/24 h or ACR ≥ 30 mg/g) Urine sediment abnormalities Electrolyte and other abnormalities due to tubular disorders Abnormalities detected by histology. Structural abnormalities detected in renal imaging. History of kidney transplantation Decreased eGFR < 60 mL/min/1.73 m^2^	Both criteria must be fulfilled for at least 3 months. Decreased eGFR < 60 mL/min/1.73 m^2^ Excluded other potential causes of kidney dysfunction. HRS-AKD should be considered when decreased eGFR maintains for less than 3 months.

AER—albumin excretion rate; ACR—urinary albumin to creatinine ratio; CKD—chronic kidney disease; HRS-CKD—hepatorenal syndrome chronic kidney disease; HRS-AKD—hepatorenal syndrome acute kidney disease; eGFR—estimated glomerular filtration rate.

**Table 5:** Summary of treatment options [161].

Form of Treatment	Grade of Recommendation
Terlipressin plus albumin	Strongly recommended, supported by evidence from randomized, controlled trials, should be considered a first-line treatment
Vasoconstrictors plus albumin	Strongly recommended for all patients
Noradrenaline plus albumin	A weaker recommendation can be considered as an alternative to terlipressin based on evidence from randomized, controlled trials
Midodrine with octreotide plus albumin	Recommended as a treatment option only when terlipressin or noradrenaline are unavailable since its efficacy is much lower than that of terlipressin
RRT	Not recommended as a standard option of treatment, the decision to initiate RRT should be based on the individual severity of HRS-AKI
LT	Strongly recommended, supported by evidence from randomized, controlled trials, as the best treatment option for all patients, regardless of their response to drug therapy
SLKT	Not recommended as a standard option of treatment and should be considered based on individual indications
Other therapies with poor evidence	
TIPS	Not recommended as a standard option of treatment
MARS	Not recommended as a standard option of treatment
FPSA	Not recommended as a standard option of treatment
TPE	Not recommended as a standard option of treatment
PDF	Not recommended as a standard option of treatment
Selepressin	Not recommended as a standard option of treatment
Serelaxin	Not recommended as a standard option of treatment
Nebivolol	Not recommended as a standard option of treatment
Pentoxifylline	Not recommended as a standard option of treatment
Rifaximin	Not recommended as a standard option of treatment
Fucoidan	Not recommended as a standard option of treatment
Stem cell therapy	Not recommended as a standard option of treatment

Abbreviations: TIPS—trans-jugular intrahepatic portosystemic shunts, RRT—renal replacement therapy, LT—liver transplant, SLKT—simultaneous liver-kidney transplantation, MARS—molecular absorbent recirculating system, FPSA—fractionated plasma separation and adsorption system, TPE—therapeutic plasma exchange, PDF—plasma diafiltration.

**Table 6:** Indications for liver transplant and MELD score [209,210].

Liver Transplant General Indications
Acute liver failure
Hepatic artery thrombosis within 14 days of liver transplant
Cirrhosis with: Decompensation (variceal bleeding, hepatic encephalopathy, or ascites) MELD score ≥ 15 Hepatopulmonary syndrome or portopulmonary hypertension (select patients)
Primary hepatic neoplasms: Hilar cholangiocarcinoma (highly selected, after neoadjuvant therapy protocol) Hepatocellular carcinoma within the Milan criteria
Inborn metabolic conditions: Cystic fibrosis with concomitant lung and liver disease Primary hyperoxaluria type I with significant renal insufficiency Familial amyloid polyneuropathy
MELD score
$MELD=9.57\times Log_{e}\left(creatinine\right)+3.78\times Log_{e}\left(totalbilirubin\right)+11.2\times Log_{e}\left(INR\right)+6.43$

Abbreviations: MELD score—Model for End-Stage Liver Disease, INR—international normalized ratio.

**Table 7:** Indications for simultaneous liver-kidney transplantation (SLKT) [165].

Simultaneous Liver-Kidney Transplantation Indications
AKI ≥ 6 consecutive weeks with one or a combination of both (weekly documentation) Dialysis eGFR/CrCl ≤ 25 mL/min
CKD with GFR ≤ 60 mL/min for >90 days with one of the following: End-stage renal disease eGFR/CrCl ≤ 30 mL/min at the time or after registration on kidney waiting list
Metabolic diseases
Safety net: Any patient who is registered on the kidney waitlist between 60 and 365 days after LT and is either on chronic hemodialysis or has an eGFR < 20 mL/min will qualify for increased priority

Abbreviations: AKI—acute kidney injury, CrCl—creatinine clearance, eGFR—estimated glomerular filtration rate, GFR—glomerular filtration rate, CKD—chronic kidney disease, LT—liver transplant.

**Table 8:** Child-Pugh scoring system [258].

Factor	1 Point	2 Points	3 Points
Encephalopathy	None	Grade I or II	Grade III or IV
Ascites	None	Slight	Moderate
Total bilirubin (mg/mL)	<2	2–3	>3
Serum Albumin (mg/mL)	>3.5	2.8–3.5	<2.8
INR (s)	<1.7	1.7–2.2	>2.2

INR—international normalized ratio of prothrombin time (PT).

## Data Availability

The data used in this article were sourced from materials mentioned in the References section.
